# Differential regulation of K_Ca_2.1 (*KCNN1*) K^+^ channel expression by histone deacetylases in atrial fibrillation with concomitant heart failure

**DOI:** 10.14814/phy2.14835

**Published:** 2021-06-10

**Authors:** Ann‐Kathrin Rahm, Teresa Wieder, Dominik Gramlich, Mara Elena Müller, Maximilian N. Wunsch, Fadwa A. El Tahry, Tanja Heimberger, Steffi Sandke, Tanja Weis, Patrick Most, Hugo A. Katus, Dierk Thomas, Patrick Lugenbiel

**Affiliations:** ^1^ Department of Cardiology Medical University Hospital Heidelberg Heidelberg Germany; ^2^ HCR (Heidelberg Center for Heart Rhythm Disorders) University Hospital Heidelberg Heidelberg Germany; ^3^ DZHK (German Centre for Cardiovascular Research), partner site Heidelberg/Mannheim University of Heidelberg Heidelberg Germany

**Keywords:** atrial fibrillation, electrophysiology, epigenetics, histone deacetylase, K_Ca_ channel

## Abstract

Atrial fibrillation (AF) with concomitant heart failure (HF) poses a significant therapeutic challenge. Mechanism‐based approaches may optimize AF therapy. Small‐conductance, calcium‐activated K^+^ (K_Ca_, *KCNN*) channels contribute to cardiac action potential repolarization. *KCNN1* exhibits predominant atrial expression and is downregulated in chronic AF patients with preserved cardiac function. Epigenetic regulation is suggested by AF suppression following histone deacetylase (HDAC) inhibition. We hypothesized that HDAC‐dependent *KCNN1* remodeling contributes to arrhythmogenesis in AF complicated by HF. The aim of this study was to assess *KCNN1* and *HDAC1–7* and *9* transcript levels in AF/HF patients and in a pig model of atrial tachypacing‐induced AF with reduced left ventricular function. In HL‐1 atrial myocytes, tachypacing and anti‐*Hdac* siRNAs were employed to investigate effects on *Kcnn1* mRNA levels. *KCNN1* expression displayed side‐specific remodeling in AF/HF patients with upregulation in left and suppression in right atrium. In pigs, *KCNN1* remodeling showed intermediate phenotypes. *HDAC* levels were differentially altered in humans and pigs, reflecting highly variable epigenetic regulation. Tachypacing recapitulated downregulation of *Hdacs* *1*, *3*, *4*, *6*, and *7* with a tendency towards reduced *Kcnn1* levels in vitro, indicating that atrial high rates induce remodeling. Finally, *Kcnn1* expression was decreased by knockdown of *Hdacs* *2*, *3*, *6*, and *7* and enhanced by genetic *Hdac9* inactivation, while anti‐*Hdac* *1*, *4*, and *5* siRNAs did not affect *Kcnn1* transcript levels. In conclusion, *KCNN1* and *HDAC* expression is differentially remodeled in AF complicated by HF. Direct regulation of *KCNN1* by HDACs in atrial myocytes provides a basis for mechanism‐based antiarrhythmic therapy.

## Graphical abstract

Atrial KCNN1 channels are remodeled in patients and pigs with atrial fibrillation. *KCNN1* remodeling is associated with changes in histone deacetylase (HDAC) expression: Inactivation of *Hdac9* enhances Kcnn1, Knock‐down of *Hdacs* 2, 3, 6, and 7 suppresses Kcnn1. *KCNN1* regulation by HDACs serves a basis for mechanism‐based antiarrhythmic therapy.

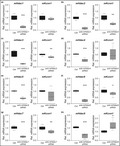

## 1 INTRODUCTION

Effective antiarrhythmic treatment of atrial fibrillation (AF) still constitutes an unmet need in cardiovascular medicine. AF underlies complex electrical and structural remodeling processes (McKinsey, 2011; Heijman et al. 2014; Nattel et al., 2020; Wijesurendra & Casadei, 2019). Concomitant heart failure (HF) worsens the prognosis of AF patients and poses a particular therapeutic challenge due to a distinct proarrhythmic atrial substrate. Atrial arrhythmogenesis in HF patients differs markedly from AF in the absence of HF. AF is characterized by shortened action potential duration (APD) (chronic (c)AF) or by no APD changes (paroxysmal (p)AF), respectively (Schmidt et al., 2015), in patients without HF. By contrast, atrial APD and effective refractory period (AERP) are prolonged in AF complicated by reduced left ventricular ejection fraction (LVEF) in humans and in animal models (Lugenbiel et al., 2017; Schmidt et al., 2017). Furthermore, atrial effective refractory period prolongation has been identified as risk factor of AF (Lee et al., 2016).

Small‐conductance, calcium‐activated K^+^ (K_Ca_, SK) channels are biophysically characterized by small unitary conductance, weak voltage‐sensitivity, and activation by intracellular Ca^2+^ (Zhang, Wu, et al., 2014). Three K_Ca_ channels (K_Ca_2.1‐3) and corresponding genes *KCNN1*‐*3* have been identified in the heart (Skibsbye et al., 2014; Tuteja et al., 2010; Xu et al., 2003; Yi et al., 2015). *KCNN1* is expressed in human and murine heart with atrial predominance (Tuteja et al., 2005; Xu et al., 2003), indicating a potential advantage for K_Ca_2.1 channels as atrial‐selective targets in AF therapy (Hancox et al., 2016). In patients with cAF and preserved LVEF, expression of K_Ca_2.1 (*KCNN1*) was reduced compared to sinus rhythm (SR) subjects (Fan et al., 2018; Yu et al., 2012). At the functional level, K_Ca_ channels underlie the cardiac *I*_K,Ca_ current and are inhibited by apamin (Zhang, Wu, et al., 2014) with different affinity (K_Ca_2.2>K_Ca_2.3>K_Ca_2.1 [Lamy et al., 2010]). Apamin‐sensitive currents have been recorded in human atrial myocytes (Skibsbye et al., 2014; Yu et al., 2012). K_Ca_ inhibition results in prolongation of atrial refractoriness and/or APD duration (Diness et al., 2010, 2011; Hsueh et al., 2013; Qi et al., 2014; Rasheed et al., 2007; Skibsbye et al., 2014, 2018; Xu et al., 2003). In rodent models, suppression of atrial arrhythmias by K_Ca_ inhibition has been described (Diness et al., 2010, 2011; Skibsbye et al., 2018). As both shortening and prolongation of atrial APD confer increased susceptibility to AF (Li et al., 2009; Qi et al., 2014; Zhang, Wu, et al., 2014; Zhang, Timofeyev, et al., 2014), the therapeutic efficacy of interventions targeting K_Ca_ channels will likely depend on achieving a delicate functional K_Ca_ homeostasis that is affected by individual patient characteristics and by environmental factors that determine specific K_Ca_ channel remodeling. However, the mechanistic basis of K_Ca_ channel remodeling in AF and HF as prerequisite for tailored, patient‐specific antiarrhythmic therapy is poorly understood.

Epigenetic regulatory mechanisms have previously been implicated in AF pathogenesis (Lkhagva et al., 2016). To date, histone deacetylase (HDAC) effects on cardiac K^+^ currents have been almost exclusively investigated using broad range inhibitors. In vitro, APD prolongation and reduced expression of K^+^ channels were observed in atrial cardiomyocytes after application of broad‐spectrum inhibitors of HDACs (Lugenbiel et al., 2018). Furthermore, inhibition of class I HDACs resulted in AF suppression in animal models (Seki et al., 2016; Skibsbye et al., 2018, Scholz et al. 2019). Very recently knockdown of Hdac2 in vitro was revealed to reduce *Kcnn3*/K_Ca_2.3 expression, corresponding to similar findings in human samples and in a porcine model of AF with concomitant HF (Rahm et al., 2021). Effects of specific HDAC isoforms on atrial *KCNN1* K^+^ ion channel expression have not been delineated in detail before. We hypothesized that *KCNN1* gene expression is regulated through epigenetic modulation in AF. *KCNN1* expression and *HDAC* remodeling were analyzed in AF patients with concomitant HF and in porcine models of atrial tachypacing‐induced AF with reduced LVEF. Furthermore, we assessed direct epigenetic effects of siRNA‐based *Hdac* inactivation on *Kcnn1* expression in atrial cells.

## 2 MATERIALS AND METHODS

### 2.1 Ethics statement

The study involving human tissue samples was conducted in accordance with the Declaration of Helsinki, and the study protocol was approved by the Ethics Committee of the University of Heidelberg (Germany; institutional approval number S‐390/2011). Written informed consent was obtained from all patients. Animal experiments have been carried out in accordance with the Guide for the Care and Use of Laboratory Animals as adopted and promulgated by the US National Institutes of Health (NIH publication No. 86‐23, revised 1985) and with EU Directive 2010/63/EU, and the current version of the German Law on the Protection of Animals was followed. Experiments involving pigs (institutional approval numbers G‐106/10 and G‐165/12) have been approved by the local animal welfare authority.

### 2.2 Patients

A total of 30 patients (51.0±12.2 years mean age; 66.7% male) with SR (*n* = 10), paroxysmal (p)AF (*n* = 10), and chronic (c)AF (i.e., persistent, long‐standing persistent or permanent AF; *n* = 10) undergoing heart transplantation due to severe HF were included (Table 1). The patient cohort with detailed characteristics has been reported previously (Lugenbiel et al., 2017, 2021; Rahm et al., 2021). Right and left atrial tissue samples were obtained from the Heidelberg CardioBiobank (Department of Cardiology, University Hospital Heidelberg) and quality controlled by the tissue bank of the National Center for Tumor Diseases (NCT) in accordance with the regulations of the tissue bank.

**TABLE 1:** Baseline characteristics of study patients

	SR (*n* = 10)	pAF (*n* = 10)	cAF (*n* = 10)
Demographics			
Men, *n* (%)	8 (80)	6 (60)	6 (60)
Age, years	50±15	52±12	51±11
Body mass index, kg/m^2^	24±3	24±5	24±5
Medical history			
Minor CAD, *n* (%)	0 (0)	5 (50)*	0 (0)
AVD, *n* (%)	2 (20)	4 (40)	0 (0)
MVD, *n* (%)	9 (90)	8 (80)	8 (80)
CAD +AVD, *n* (%)	0 (0)	3 (30)	0 (0)
ICM, *n* (%)	4 (40)	2 (20)	3 (30)
DCM, *n* (%)	6 (60)	8 (80)	4 (40)
PPCM, *n* (%)	0 (0)	0 (0)	1 (10)
Amyloidosis, *n* (%)	0 (0)	0 (0)	2 (20)
Hypertension, *n* (%)	4 (40)	5 (50)	3 (30)
Diabetes, *n* (%)	4 (40)	5 (50)	2 (20)
Hyperlipidemia, *n* (%)	3 (30)	4 (40)	4 (40)
Echocardiography			
LA size (mm)	47±3	51±7	49±9
LVEF (%)	19±6	21±9	17±9
Medication			
Amiodarone, *n* (%)	4 (40)	3 (30)	3 (30)
Ivabradine, *n* (%)	3 (30)	0 (0)	2 (20)
Digitalis, *n* (%)	3 (30)	5 (50)	7 (70)
ACE inhibitors, *n* (%)	8 (80)	6 (60)	7 (70)
AT1 blockers, *n* (%)	1 (10)	2 (20)	1 (10)
Βeta‐blockers, *n* (%)	9 (90)	10 (100)	8 (80)
Diuretics, *n* (%)	9 (90)	10 (100)	10 (100)
Nitrates, *n* (%)	0 (0)	0 (0)	0 (0)
Lipid‐lowering drugs, *n* (%)	6 (60)	4 (40)	4 (40)
OAC, *n* (%)	8 (80)	7 (70)	6 (60)

Please note that patient characteristics have been published previously (Lugenbiel et al., 2021, 2017; Rahm et al., 2021). Statistical comparisons between pAF / cAF versus SR groups were performed using ANOVA followed by Bonferroni correction for continuous variables and chi‐square tests for categorical variables.

Abbreviations: ACE, angiotensin converting enzyme; AT, angiotensin receptor; AVD, aortic valve disease; CAD, coronary artery disease (minor concomitant CAD, not explaining severe LVEF reduction); cAF, chronic atrial fibrillation; DCM, dilated cardiomyopathy; ICM, ischemic cardiomyopathy (including severe CAD); LA, left atrial; LVEF, left ventricular ejection fraction; MVD, mitral valve disease; OAC, oral anticoagulation; pAF, paroxysmal atrial fibrillation; PPCM, peripartum cardiomyopathy; SR, sinus rhythm.

* *p* < 0.05 versus SR.

### 2.3 Human tissue processing

Human heart samples were immediately dissected in the operating room following explanation of the recipient's heart during cardiac transplantation. Atrial tissue sections were shock‐frozen in liquid nitrogen and stored at −80°C. An uninterrupted cooling chain was maintained prior to molecular analyses.

### 2.4 AF animal model

AF‐associated remodeling of HDACs and K_Ca_2.1 channels was assessed using a porcine AF model (Lugenbiel et al., 2017). AF was induced in domestic swine by rapid atrial burst pacing *via* an implanted cardiac pacemaker located in the right atrium (RA). Owing to rapid ventricular rate response during AF, pigs subjected to high‐rate atrial pacing displayed reduced LV function. Animals carrying inactive pacemakers served as controls. Cardiac samples analyzed in this work were obtained from previously reported pigs 7 days (*n* = 5) (Lugenbiel et al., 2015) (ethics approval number G‐106/10) or 14 days (*n* = 5) (Lugenbiel et al., 2017) (ethics approval number G‐165/12) after the initiation of atrial burst pacing or from corresponding control pigs not subjected to AF induction (*n* = 5 each). The ARRIVE (Animal Research: Reporting of In Vivo Experiments) guidelines were observed when reporting the use of animals. Please refer to prior publications for full experimental procedures and related information (Lugenbiel et al., 2015, 2017). Echocardiographic and electrophysiological data have been reported previously (Lugenbiel et al., 2015, 2017).

### 2.5 Protein isolation and Western blotting

Porcine atrial tissues were lysed and homogenized as reported previously (Rahm et al., 2021). Briefly, protein concentration was determined using the bicinchoninic acid protein assay (Thermo Scientific), and proteins were diluted to equal concentrations with sterile water. Protein immunodetection was performed by sodium dodecyl sulfate (SDS) gel electrophoresis with 10% SDS–polyacrylamide gels and Western blotting with polyvinylidene difluoride membranes. Loading of equal amounts of protein was additionally confirmed with Ponceau Red staining (data not shown). Membranes were blocked with 5% milk in PBS‐T for 2 h at room temperature and developed using primary antibodies directed against K_Ca_2.1 (1:1,000; APC‐039, Alomone Labs). For controls, the respective control peptide for K_Ca_2.1 supplied by the company was used (data not shown). Horseradish peroxidase‐conjugated donkey anti‐rabbit (ab6802; Abcam) secondary antibody was used. Signals were developed using the enhanced chemiluminescence assay (ECL Western Blotting Reagents, GE Healthcare). After removal of antibodies (ReBlot Strong Stripping Solution), the membranes were re‐probed with anti‐glyceraldehyde 3‐phosphate dehydrogenase (GAPDH) (1:10,000, ab181602, Abcam; or 1:20,000, G8140‐01, Biomol) antibodies and corresponding secondary antibodies (ab6802, Abcam; or 1031–05, Southern Biotech). Protein content was normalized to respective control samples for quantification of optical density (ImageJ 1.50i Software, National Institutes of Health).

### 2.6 HL‐1 cell culture and siRNA transfection

HL‐1 cardiac muscle cells derived from the AT‐1 mouse atrial myocyte tumor lineage were provided earlier by Dr. William Claycomb (Louisiana State University Health Science Center). K_Ca_ channel expression in HL‐1 cells has been demonstrated previously (Yi et al., 2015). Cells were cultured in supplemented Claycomb medium (Sigma‐Aldrich). SiRNAs directed against HDAC1 (sc‐29344), HDAC2 (sc‐29346), HDAC3 (sc‐35539), HDAC4 (sc‐35541), HDAC5 (sc‐35543), HDAC6 (sc‐35545), HDAC7 (sc‐35545), and HDAC9 (sc‐35551) were obtained from Santa Cruz Biotechnology. Transfections were performed using lipofectamine RNAiMax (ThermoFisher Scientific) according to the manufacturer's instructions.

### 2.7 Electrical stimulation of HL‐1 cells

Gelatin‐/fibronectin‐coated six‐well dishes were seeded with 4–5 × 10^6^ HL‐1 cells. Cells were ≥90% confluent after 24 h incubation and subjected to electrical stimulation as described (Lugenbiel et al., 2018) using the C‐Pace EP system (IonOptix). Stimulation was performed with 10 V/10 ms pulses at 4 Hz rates. Cell viability was visually assessed by microscopic examination following rapid electrical stimulation for 24 h, before cells were harvested and RNA was isolated. Control cells not subjected to stimulation were otherwise handled and maintained similarly.

### 2.8 Quantitative real‐time PCR

Quantitative real‐time PCR (RT‐qPCR) was carried out with the 7500 Fast Real‐Time PCR System (Applied Biosystems) as reported (Lugenbiel et al., 2017, 2018). Total RNA was isolated from indicated human and porcine cardiac regions and from HL‐1 cells using TRIzol‐Reagent (Invitrogen). Digestion of genomic DNA was performed with the TurboDNase‐Kit (Thermo Fisher Scientific) according to the manufacturer's instructions. DNA synthesis was performed by reverse transcription with the Maxima First Strand cDNA Synthesis Kit for RT‐qPCR (Thermo Fisher Scientific) using 3 µg of total RNA. Optical detection plates (96 wells; Applied Biosystems) were loaded to a total volume of 10 µl per well, consisting of 0.5 µl cDNA, 5 µl TaqMan Fast Universal Master Mix (Applied Biosystems), and 6‐carboxyfluorescein (FAM)‐labeled TaqMan probes and primers (TaqMan Gene Expression Assays; Applied Biosystems) (Table 2). For detection of porcine *HDACs* *1*, *2*, *3*, *6*, *7*, and *9*, CYBR green qRT‐PCR was performed using Power SYBR Green PCR Master Mix (Applied Biosystems) and appropriate primers (50 µM; Table 2). Of note, selective primers for CYBR green qRT‐PCR of porcine *HDAC5* could not be identified. In addition, predesigned primers and probes detecting species‐specific *GAPDH* were used for normalization. All qRT‐PCR reactions were performed in duplicates or higher replicates, and non‐template controls and dilution series were included on each plate for quantification. Data analyses were performed using the second derivative method.

**TABLE 2:** TaqMan assays and CYBR‐green primers used for real‐time quantitative polymerase chain reactions

Target gene	Human	Porcine	Murine
*KCNN1*	Hs01109326_m1	AJLJI7X*	Mm01349167_m1
*HDAC1*	Hs02621185_s1	F: 5‘‐CAAGCCGGTCATGTCCAAAG R: 5‘‐ACCTAACCGATCCCCAGACA	Mm02391771_g1
*HDAC2*	Hs00231032_m1	F: 5‘‐ACAGTCAAAGGTCACGCTAA R: 5‘‐AGCTTGAAGTCCGGTCCAAA	Mm00515108_m1
*HDAC3*	Hs00187320_m1	F: 5‘‐GAGAATTACAGCAGGCCGGA R: 5‘‐GGCAAGCCCAGTCAGTCTTA	Mm00515916_m1
*HDAC4*	Hs01041648_m1	AJ70L9G*	Mm01299557_m1
*HDAC5*	Hs00608351_m1	n/a	Mm01246076_m1
*HDAC6*	Hs00997427_m1	F: 5‘‐GCTTGCTTGCTTGCACTCTT R: 5‘‐CCTGACGCGGTTCTTAGGAG	Mm00515945_m1
*HDAC7*	Hs01045864_m1	F: 5‘‐CGGGAGCTCAAGAACGGTTT R: 5‘‐CCATGGCTGTGGAATGGTCT	Mm00469527_m1
*HDAC9*	Hs01081558_m1	F: 5‘‐ACAACAGAACGGATGGGGTG R: 5‘‐GTCCACCACAGGCATCATCA	Mm01293999_m1
*GAPDH*	Hs02786624_g1	Ss03375629_u1 F: 5‘‐GTCGGAGTGAACGGATTTGGC R: 5‘‐CTTGCCGTGGGTGGAATCAT	Mm99999915_g1

DNA sequences for CYBR green primers are displayed. Custom‐designed TaqMan assays are indicated by an asterisk.

### 2.9 Statistics

Continuous patient data are provided as mean±standard deviation, and categorical variables are given as frequency and percentage. Experimental data are expressed as box plots with dots representing individual data points. Statistical differences of continuous variables were determined with Origin software (OriginLab) using unpaired Student's *t*‐tests (two‐sided tests). Categorical data were analyzed using the chi‐square test. *p* < 0.05 was considered statistically significant. Multiple comparisons were performed using one‐way ANOVA. If the hypothesis of equal means could be rejected at the 0.05‐level, pair wise comparisons of groups were made and the probability values were adjusted for multiple comparisons using the Bonferroni correction.

## 3 RESULTS

### 3.1 Side‐specific remodeling of atrial *KCNN1* expression in human AF patients with concomitant HF

**FIGURE 1:**
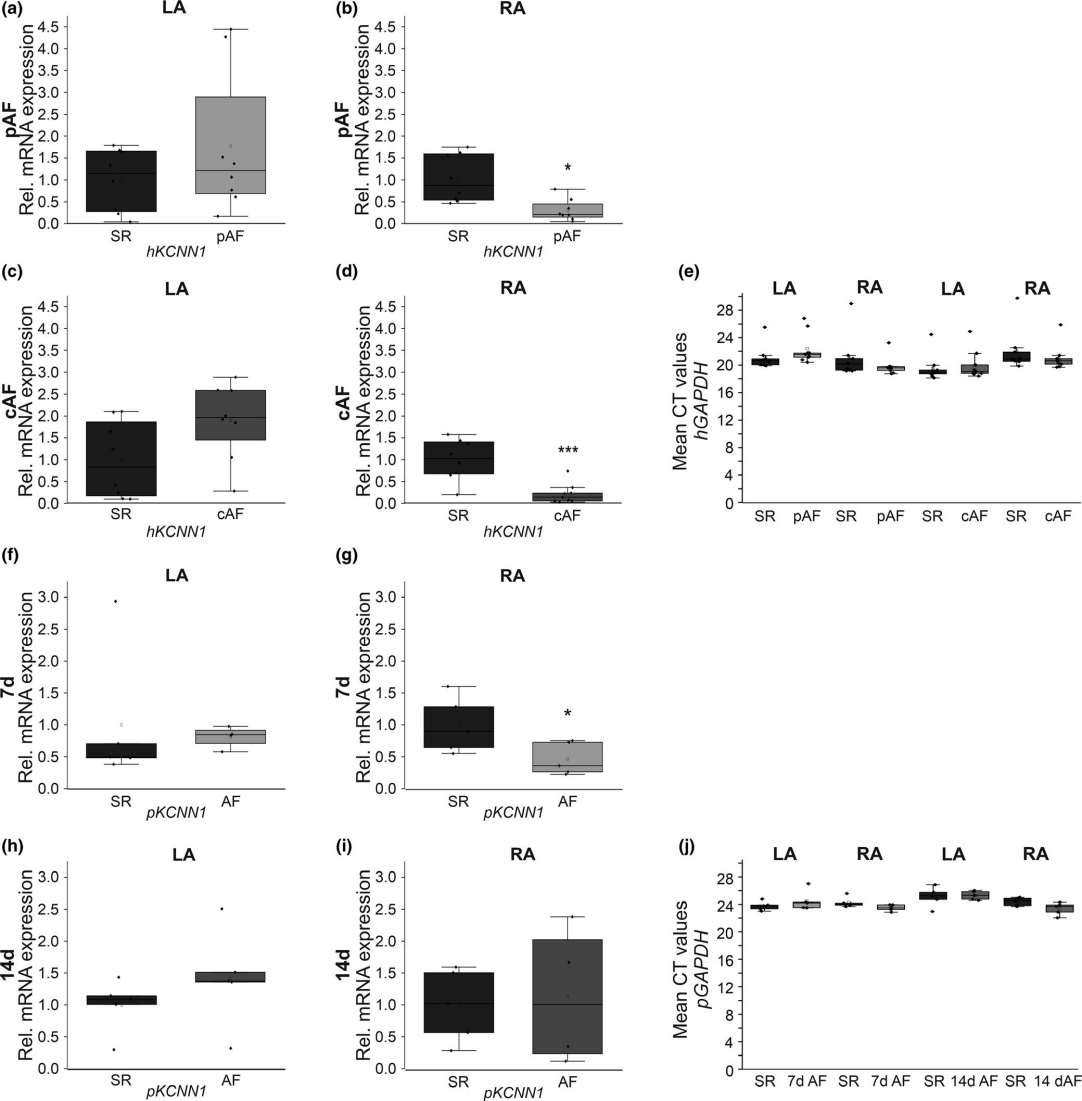
Remodeling of K_Ca_2.1 channels associated with AF in HF patients and a porcine AF model. K_Ca_2.1 (*KCNN1*) channel transcript levels were assessed in patients with paroxysmal AF (pAF; *n* = 10, a, b) or chronic AF (cAF; *n* = 10, c, d) and are displayed for left atrium (LA; a, c) and right atrium (RA; b, d) in comparison to sinus rhythm patients (SR; *n* = 10). (e) Mean threshold cycle (CT) levels of the housekeeping gene glyceraldehyde 3‐phosphate dehydrogenase (*GAPDH*) in human left (LA) and right atria (RA) (*n* = 10 each). (f–i) K_Ca_2.1 (*KCNN1*) mRNA levels obtained from AF pigs after 7 days (*n* = 5, f, g) or 14 days of atrial burst pacing (*n* = 5, h, i) are provided compared to respective SR controls (SR; *n* = 5 each). (f, g) LA, left atrium. (h, i) RA, right atrium. (j) Mean porcine *GAPDH* CT levels in LA and RA tissue (*n* = 5 each). Data are provided as box plots with underlying dots indicating original data; *p < 0.05, ***p < 0.001 versus SR controls

To assess K_Ca_2.1 channel remodeling in human AF and HF, *KCNN1* mRNA expression was analyzed in left atrium (LA) and right atrium (RA) of study patients. Paroxysmal AF (pAF) was associated with numerically increased *KCNN1* levels in human LA (+78%, *n* = 10, *p* = 0.31) and significantly suppressed *KCNN1* mRNA abundance in RA (−73%, *n* = 10, *p* = 0.010) tissue, respectively (Figure 1a,b). In patients with cAF *KCNN1* expression was similarly increased by 91% in LA (*n* = 10, *p* = 0.074) and decreased by 78% in RA (*n* = 10, *p* < 0.0001) compared to individuals with SR (*n* = 10 each) (Figure 1c,d). Mean human *GAPDH* levels were not significantly different between AF patients and SR controls (pAF vs. SR: *p* = 0.20 [LA], *p* = 0.32 [RA]; cAF vs. SR: *p* = 0.64 [LA], *p* = 0.43 [RA]; Figure 1e).

### 3.2 Differential remodeling of atrial *KCNN1* levels in porcine AF/HF models

Changes in *KCNN1* suppression were recapitulated in an established porcine AF model with concomitant reduction of LV function. Tissue samples were obtained from previously described animals subjected to AF/HF induction via repetitive atrial burst pacing by an implanted cardiac pacemaker for 7 days (*n* = 5; Figure 1f,g [Lugenbiel et al., 2015]) or 14 days (*n* = 5; Figure 1h,i [Lkhagva et al., 2016]). Corresponding animals carrying inactive pacemakers reported earlier served as controls (*n* = 5 each [Lugenbiel et al., 2015; Lugenbiel et al., 2017]). AF/HF was associated with significant downregulation of *KCNN1* mRNA levels in RA tissue by 53% (*p* = 0.038) compared to SR controls (Figure 1g). By contrast, mean *KCNN1* mRNA abundance in the LA (−19%, *p* = 0.79) was not altered (Figure 1f). In addition, no significant reduction in *KCNN1* transcript abundance was detected after 14 days AF (Figure 1h,i). Rather, *KCNN1* mRNA expression was numerically increased in porcine LA (+42%, *n* = 10, *p* = 0.53) and RA (+13%, *n* = 10, *p* = 0.76) tissue, respectively. There were no significant differences between mean *GAPDH* levels of AF animals and SR controls (SR vs. 7 days AF: *p* = 0.33 [LA], *p* = 0.09 [RA]; SR vs. 14 days AF: *p* = 0.79 [LA], *p* = 0.08 [RA]; Figure 1j).

**FIGURE 2:**
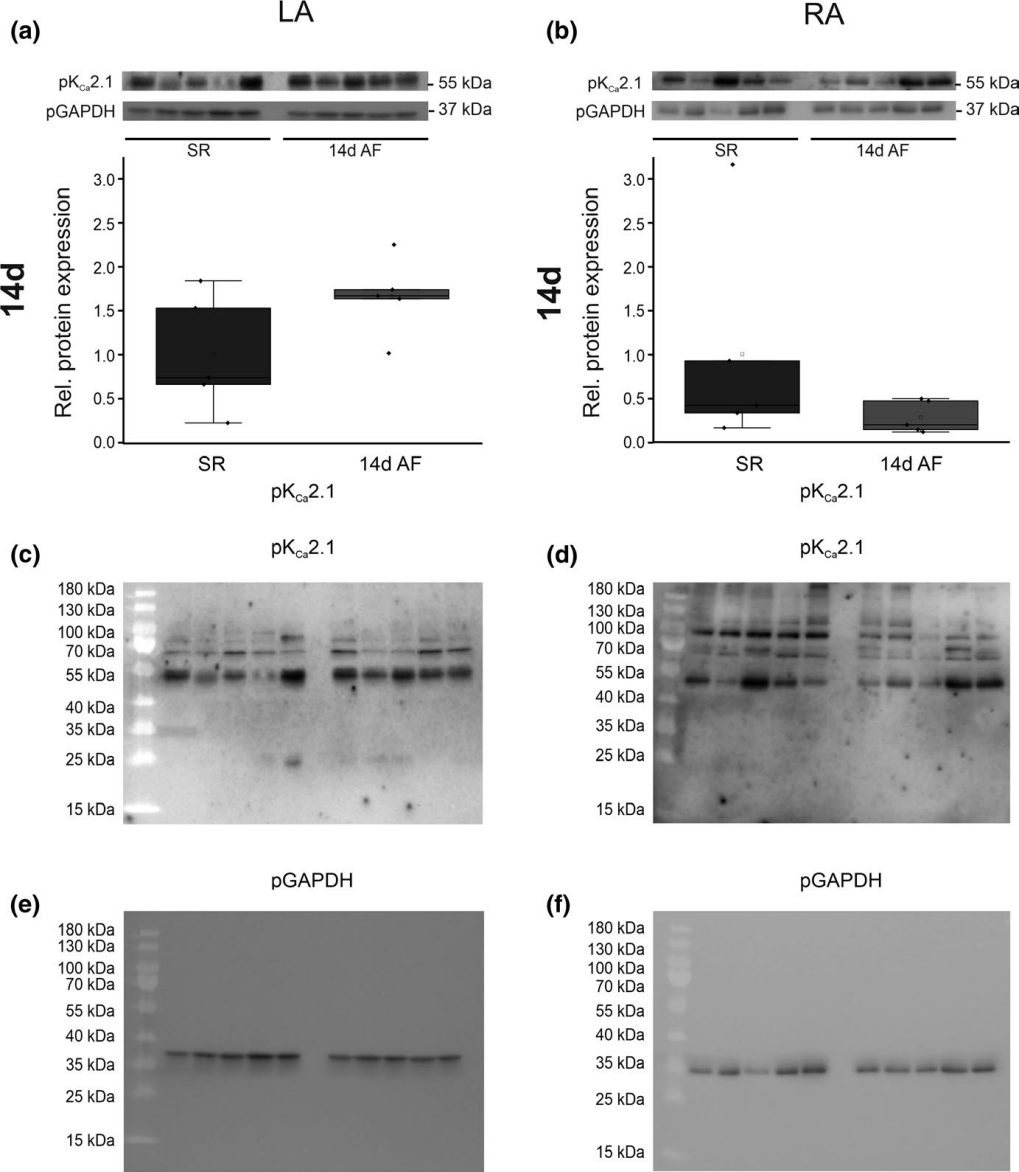
Expression of K_Ca_2.1 channel protein in a porcine atrial fibrillation (AF)/heart failure model. K_Ca_2.1 protein levels assessed in AF pigs after 14 days of atrial burst pacing (14 days AF; *n* = 5) are provided compared to SR controls (SR; *n* = 5). Data are expressed as box plots with underlying dots indicating original data relative to SR controls. (a) LA, left atrium. (b) RA, right atrium. For reference, full blots incubated with anti‐K_Ca_2.1 antibodies (c, d) and anti‐GAPDH antibodies (e, f) are shown, corresponding to cropped pictures displayed in panels (a and b)

Protein analyses were confined to animals studied after 14 days atrial burst pacing due to limited sample availability (Figure 2). AF was associated with numerical K_Ca_2.1 protein expression increase in LA (+66%, *n* = 10, *p* = 0.10) and decrease in RA tissue (−72%, *n* = 10, *p* = 0.26), respectively, without achieving statistical significance. Porcine GAPDH protein levels quantified in LA (*p* = 0.054) or RA (*p* = 0.882) tissue were not significantly different between SR and AF groups.

### 3.3 Atrial *HDAC* expression alterations in AF/HF patients

**FIGURE 3:**
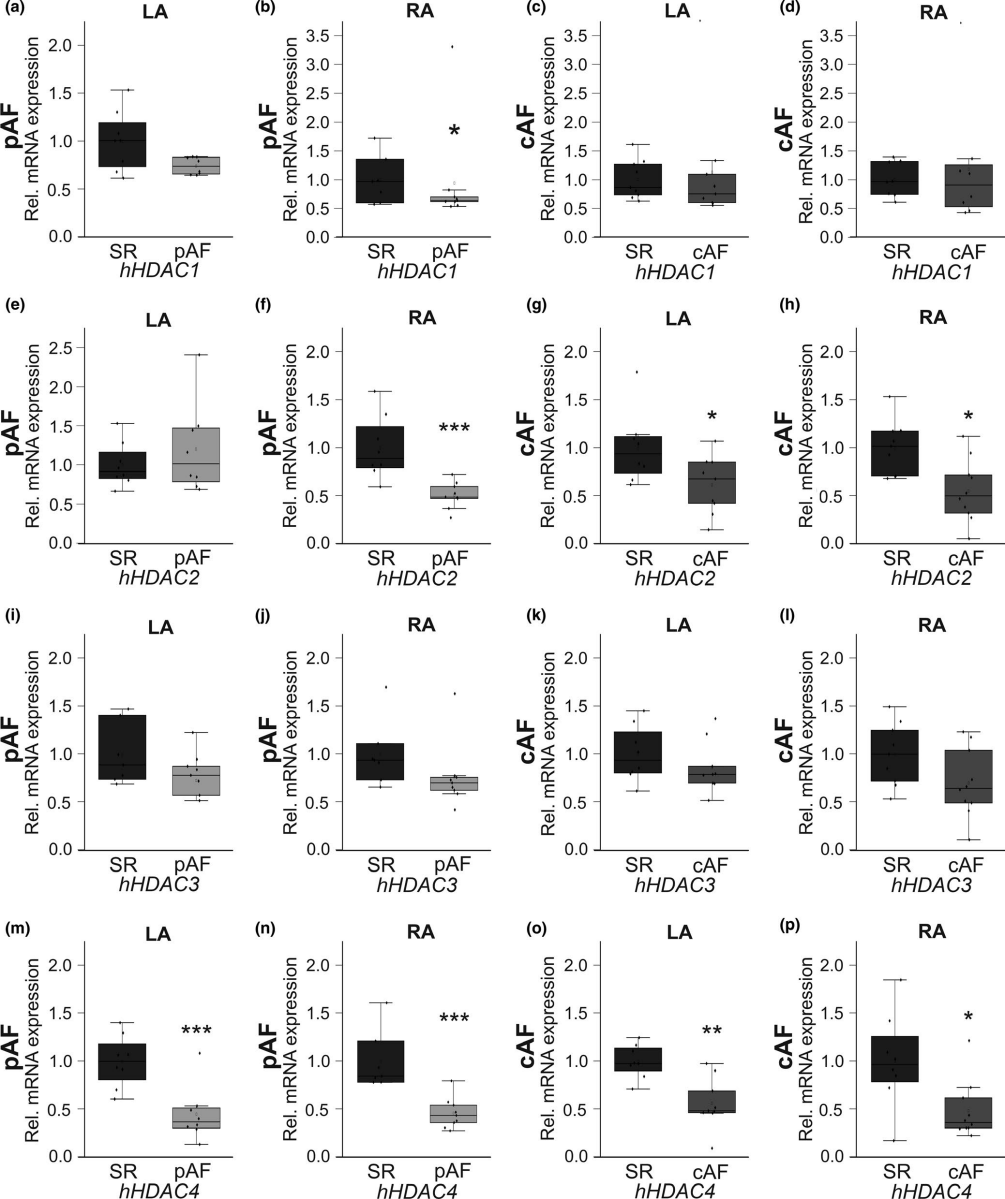
AF‐associated remodeling of *HDAC1*, *2*, *3*, and *4* transcripts in HF patients. *HDAC* mRNA levels were measured in patients with paroxysmal AF (pAF; *n* = 10, a, b, e, f, i, j, m, n) or chronic AF (cAF; *n* = 10, c, d, g, h, k, l, o, p) compared with HF patients in sinus rhythm (SR; *n* = 10). Tissue was acquired from left atrium (LA; a, c, e, g, i, k, m, o) and right atrium (RA; b, d, f, h, j, l, n, p), respectively. Please note that data on *HDAC2* have been published previously (Rahm et al., 2021). Data are given as box plots, dots represent single data points; *p < 0.05, **p < 0.01, ***p < 0.001 versus SR patients

**FIGURE 4:**
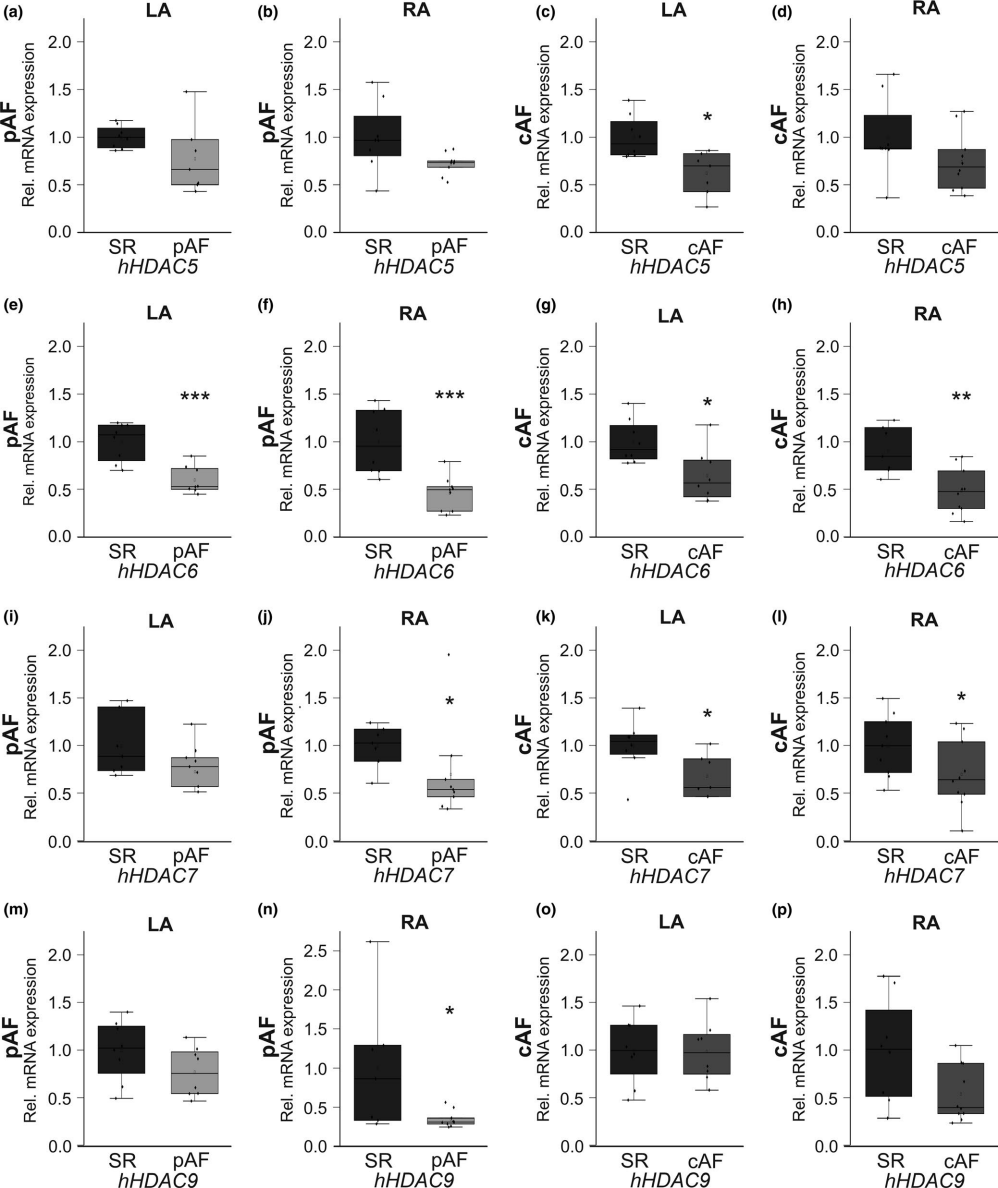
AF‐related changes in *HDAC5*, *6*, *7*, and *9* mRNA expression in HF patients. *HDAC* transcript levels were assessed in patients with paroxysmal AF (pAF; *n* = 10, a, b, e, f, i, j, m, n) or chronic AF (cAF; *n* = 10, c, d, g, h, k, l, o, p) in comparison to HF patients in sinus rhythm (SR; *n* = 10). Tissue samples were obtained from left atrium (LA; a, c, e, g, i, k, m, o) and right atrium (RA; b, d, f, h, j, l, n, p), respectively. Data are given as box plots, dots represent single data points; *p < 0.05, **p < 0.01, ***p < 0.001 versus SR patients

To evaluate a potential role of HDACs in K_Ca_2.1 channel remodeling, HDAC transcript levels were next investigated in the AF/HF patient cohort. Analyses comprising *HDAC*s 1, 2, 3, 4, 5, 6, 7, and 9 were performed stratified by region (LA vs. RA) and by rhythm status (pAF vs. cAF) and compared to individuals with SR (*n* = 10 each). The data are summarized in Figure 3 (a–p; *HDAC*s 1–4) and Figure 4 (a‐p; *HDAC*s 5–7 and 9). *HDAC*s 4 and 6 were uniformly reduced in LA and RA tissue of pAF and cAF patients. *HDAC4* was suppressed in pAF patients (LA, −55%, *p* < 0.0001; RA, −54%, *p* < 0.0001) and cAF cases (LA, −49%, *p* = 0.007; RA, −52%, *p* = 0.038) (Figure 3m–p). In addition, global AF‐related *HDAC6* downregulation was reflected by reduced mRNA levels among study subjects with pAF (LA, −40%, *p* < 0.0001; RA, −54%, *p* < 0.0001) and cAF cases (LA, −36%, *p* = 0.011; RA, −47%, *p* = 0.003), respectively (Figure 4e–h). More selective remodeling was observed with HDACs 1, 2, 5, 7 and 9. *HDAC2* mRNA abundance was reduced in RA (−49%, *p* = 0.0005) of pAF patients and in LA and RA of cAF cases (LA, −38%, *p* = 0.033; RA, −45%, *p* = 0.018) (Figure 3f–h). *HDAC7* suppression similarly affected RA of pAF subjects (−30%, *p* = 0.033) and both LA and RA of cAF patients (LA, −37%, *p* = 0.028; RA, −35%, *p* = 0.030) (Figure 4i–l). Remodeling of *HDACs* 1, 5, and 9 occurred in more specific fashion. *HDAC1* was reduced in RA tissue of pAF patients by 38% (*p* = 0.021) (Figure 3b), while HDAC5 suppression was limited to LA of cAF subjects (−30%, *p* = 0.030) (Figure 4c). Finally, *HDAC9* transcript levels were downregulated in RA of pAF subjects (−65%, *p* = 0.046), whereas numerical reduction of LA *HDAC9* mRNA by 45% did not reach statistical significance (*p* = 0.061) (Figure 4n,p). The remaining analyses did not reveal statistically significant results.

### 3.4 *HDAC* remodeling in pigs exhibiting AF and HF

**FIGURE 5:**
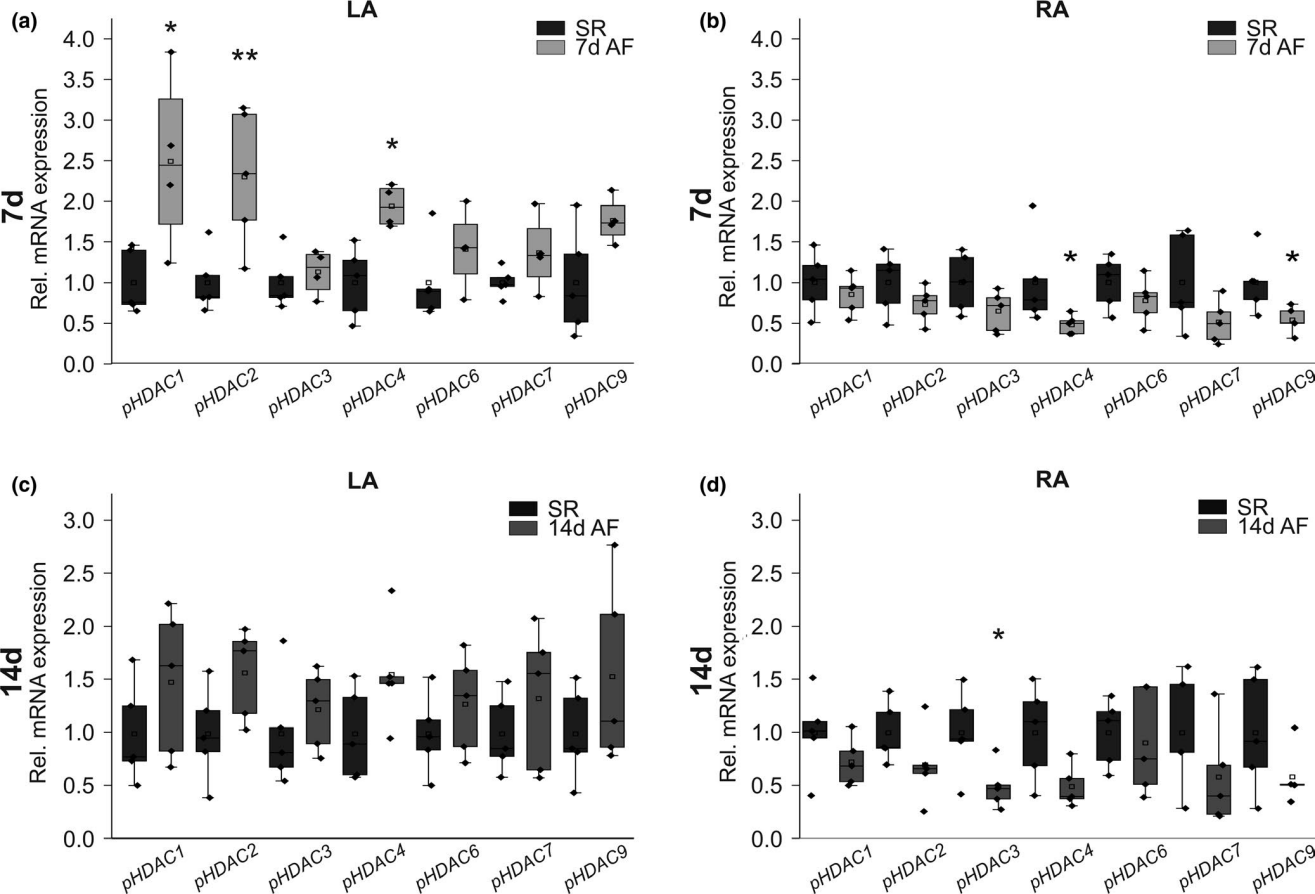
Transcriptional regulation of *HDACs* in pig models of AF/HF. Messenger RNA expression of indicated *HDACs* was studied in *n* = 5 pigs per group exhibiting AF triggered by atrial burst pacing (AF) for 7 days (a, b) or 14 days (c, d) in comparison with corresponding control animals showing sinus rhythm (SR; *n* = 5 each). (a, c) LA, left atrium. (b, d) RA, right atrium. Please note that data on *HDAC2* transcript levels in pigs have been published previously (Lugenbiel et al., 2021; Rahm et al., 2021). *p < 0.05, **p < 0.01 versus SR animals

In pig models of AF and HF we detected region‐specific *HDAC* remodeling (Figure 5a–d). HDACs tended to be upregulated in LA tissue (Figure 5a,c) and downregulated in RA samples (Figure 5b,d) after 7 days and 14 days of AF. Side‐specific remodeling was confirmed 7 d after the initiation of atrial tachypacing by significant increases in LA tissue observed with *HDAC*s 1 (+149%, *p* = 0.023), 2 (+130%, *p* = 0.010), and 4 (+94%, *p* = 0.025) (Figure 5a), and by decreased mRNA levels of *HDAC*s 4 (−52%, *p* = 0.036) and 9 (−46%, *p* = 0.025) in RA samples (Figure 5b). Furthermore, RA *HDAC3* transcript abundance was reduced by 50% (*p* = 0.041) in the 14‐day animal group (Figure 5d). Please note that *HDAC5* expression could not be analyzed in porcine tissue as the identification of specific primers was not feasible. The localization of the pacing electrode in the RA of study pigs subjected to AF induction suggests a role for rapid atrial electrical activity as potential mechanistic trigger of differential RA remodeling (Lugenbiel et al., 2017) that was assessed next in vitro.

### 3.5 Suppression of *Kcnn1* and *Hdacs* by rapid atrial pacing in murine HL‐1 atrial cells

**FIGURE 6:**
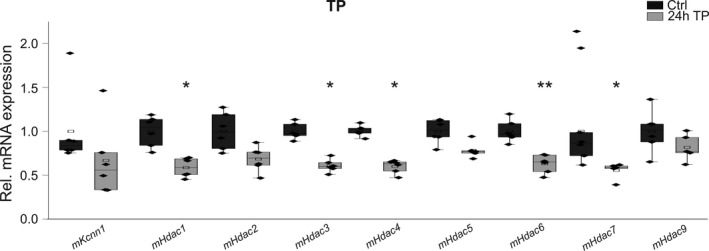
Effects of electrical tachypacing (TP) on mRNA levels in atrial HL‐1 myocytes. Remodeling of *Kcnn1* potassium channel and *Hdac* mRNA expression in HL‐1 cells subjected to electrical TP. Transcript levels of indicated Hdacs were calculated relative to untreated controls (Ctrl) as box plots with single dots representing individual data values (*n* = 6 each). Data on *HDAC2* have been shown previously (Rahm et al., 2021). *p < 0.05, **p < 0.01 versus control HL‐1 cells not subjected to tachypacing

To test whether high‐rate electrical activity triggers downregulation of *KCNN1* and *HDAC*s, cultured HL‐1 atrial myocytes were subjected to rapid electrical pacing (TP) for 24 h (Figure 6). TP induced a tendency toward *Kcnn1* mRNA suppression by 33% (*n* = 6; *p* = 0.11) compared to non‐paced control cells (*n* = 6) (Figure 6). In addition, rapid electrical pacing triggered significant transcriptional downregulation of *Hdacs* *1* (−41%, *n* = 6; *p* = 0.025), *3* (−39%, *n* = 6; *p* = 0.020), *4* (−40%, *n* = 6; *p* = 0.013), *6* (−37%, *n* = 6; *p* = 0.009), and 7 (−44%, *n* = 6; *p* = 0.023), respectively (Figure 6).

### 3.6 Modulation of *Kcnn1* expression after genetic *Hdac* knockdown in atrial myocytes

**FIGURE 7:**
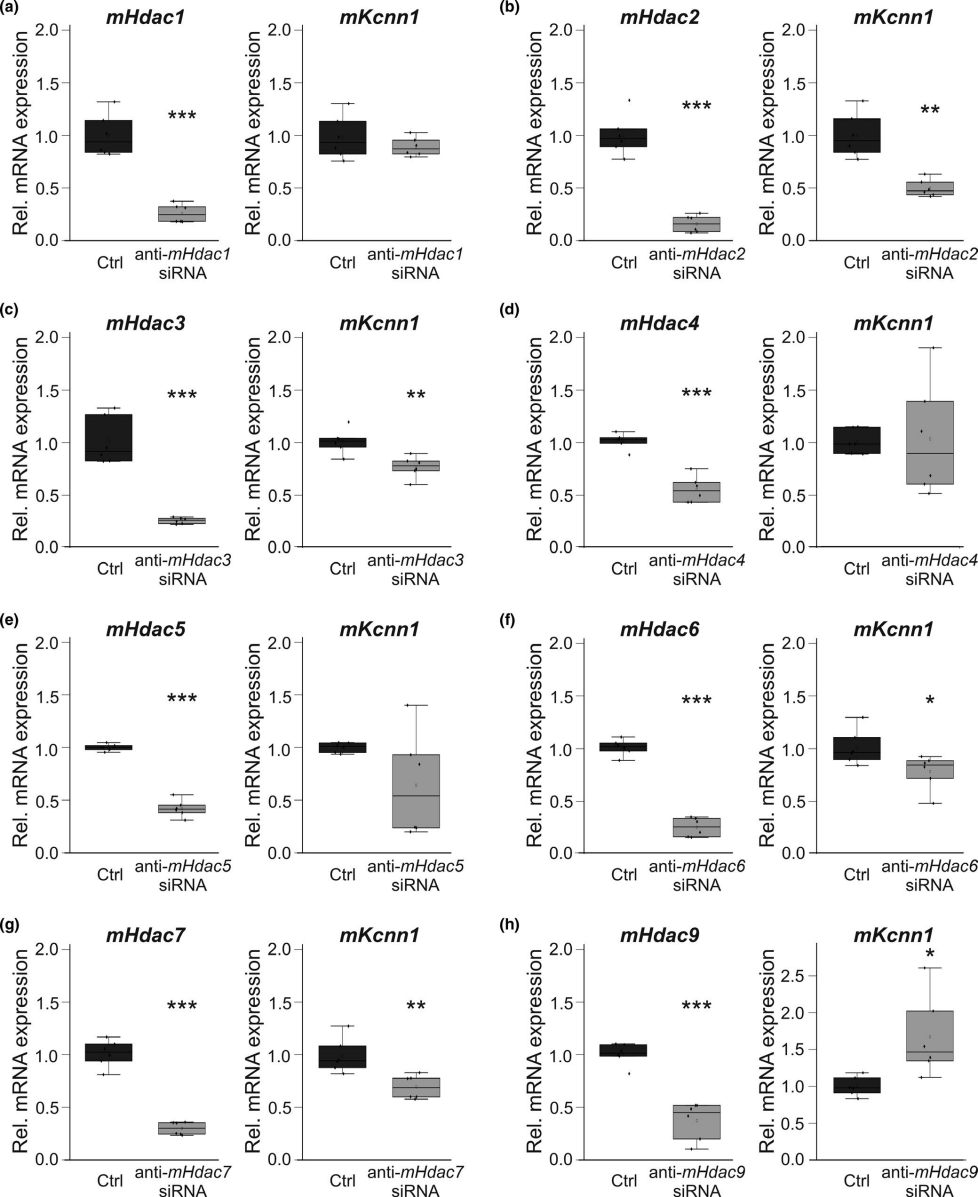
*Hdac*‐related remodeling of *Kcnn1* mRNA in HL‐1 atrial myocytes. (a–h) Left panels, transcript abundance of indicated Hdacs was calculated following application of respective anti‐*Hdac* siRNAs (*n* = 6 each) in relation to control cell cultures (Ctrl; *n* = 6 each). (a‐h) Right panels, relative *Kcnn1* (*n* = 6 each) mRNA levels were measured after genetic knockdown of indicated Hdacs and compared with respective controls in the absence of siRNA (*n* = 6 each). Please note that data on *Hdac2* suppression by anti‐*Hdac2* siRNA have been published previously (Rahm et al., 2021). Data are given as box plots with dots representing individual data. *p < 0.05, **p < 0.01, ***p < 0.001 versus control cells

Changes in *HDAC* and *KCNN1* transcript levels in patients and pigs with AF complicated by HF indicate epigenetic regulation of *KCNN1* expression. To provide evidence for direct effects of HDACs on K_Ca_2.1 channel mRNA expression, we selectively applied anti‐*Hdac* siRNAs to HL‐1 atrial cells. First, effective *Hdac* knockdown was established by reduction of *Hdacs* *1* (−74%, *n* = 6; *p* < 0.0001), *2* (−84%, *n* = 6; *p* < 0.0001), *3* (−76%, *n* = 6; *p* = 0.0004), *4* (−46%, *n* = 6; *p* < 0.0001), *5* (−58%, *n* = 6; *p* < 0.0001), *6* (−76%, *n* = 6; *p* < 0.0001), *7* (−71%, *n* = 6; *p* < 0.0001), and *9* (−63%, *n* = 6; *p* < 0.0001) compared to untreated cells after incubation with anti‐*Hdac* siRNA, respectively (Figure 7a–h). *Kcnn1* mRNA abundance was decreased via genetic inactivation of *Hdac*s *2* (‐ 50%, *n* = 6; *p* = 0.001), *3* (−24%, *n* = 6; *p* = 0.003), *6* (−23%, *n* = 6; *p* = 0.037), and *7* (−30%, *n* = 6; *p* = 0.005) (Figure 7b,c,f,g). By contrast, *Kcnn1* transcript levels were numerically enhanced following knockdown of *Hdac9* (+67%, *n* = 6; *p* = 0.29) (Figure 7h).

## 4 DISCUSSION

### 4.1 AF complicated by HF is characterized by distinct alterations in K_Ca_2.1 (*KCNN1*) channel expression

*KCNN1* expression is differentially remodeled in patients and animal models of AF and HF. In humans with severely impaired left ventricular function, pAF and cAF are associated with side‐specific *KCNN1* expression changes. We found *KCNN1* upregulation in LA and downregulation in RA tissue, indicating spatial specificity of regulatory pathways within a specific cellular, biochemical, genetic, and epigenetic background. In a porcine model characterized by a combined phenotype of AF with tachycardia‐induced impairment of left ventricular function, AF/HF‐dependent atrial *KCNN1* remodeling exhibited temporal in addition to spatial differences. After 7 days of tachypacing, left atrial *KCNN1* levels were not markedly changed, whereas *KCNN1* transcripts were less abundant in RA compared to SR controls. By contrast, left and right atrial *KCNN1* showed a tendency towards increased levels after 14 days, reflecting a shift in the balance of pathways regulating *KCNN1* expression. The complex picture of *KCNN1* remodeling in AF complicated by HF is most likely the result of multiple mechanisms that are differentially active in right versus LA and in paroxysmal versus chronic forms of AF, with additional stimuli generated by the degree of left ventricular dysfunction in HF. The regulatory pathways that interact to achieve a delicate *KCNN1* levels homeostasis are currently not known. Thus, we explored epigenetic, HDAC‐dependent mechanisms in more detail.

### 4.2 AF and HF are linked to variable atrial *HDAC* remodeling

The present work advances the current understanding of the role of histone modification in cardiac arrhythmogenesis and antiarrhythmic therapy. Based on the analyses of human atrial tissue and samples obtained from the pig model, HDACs display subtype‐specific remodeling that is affected by disease stages in humans (pAF vs. cAF) and by different AF duration (7 d vs. 14 d) in pigs. Furthermore, spatial differences between RA and LA were observed. These were pronounced in the pig model that exhibited HDAC remodeling that appeared to be particularly sensitive to the proarrhythmic stimulus, right atrial tachypacing. Indeed, high‐rate atrial pacing of HL‐1 atrial cells in vitro induced (at least numerical) downregulation of most *Hdacs* investigated, which is consistent with reduced *HDAC* mRNA levels in vivo in right atria of pigs (i.e., in close spatial relation to the pacing electrode) compared to more remote left atria. This approach revealed a direct mechanistic role for high atrial rates in epigenetic remodeling and AF pathophysiology. In addition, the genetic and epigenetic background in RA tissue may differ from LA, resulting in different directions of *HDAC* (and *KCNN*) remodeling despite similar stimuli. High variability of HDAC remodeling in humans and pigs provides a basis for differential regulation of HDAC‐dependent pathways in different AF/HF stages, including modulation of *KCNN1* expression in AF patients with concomitant HF. Of note, reduced *Hdac6* transcript levels in pAF or cAF patients with concomitant LV dysfunction as well as in HL‐1 cells subjected to 24 h of tachypacing are in contrast to increased *Hdac6* protein levels reported previously in the same in vitro system after 4–8 h tachypacing and in AF patients with undisclosed LV function (Zhang, Wu, et al., 2014). This observation highlights additional regulators affecting HDAC remodeling.

### 4.3 Genetic *HDAC* inactivation induces remodeling of *KCNN1* expression in subtype‐dependent fashion

In a specific experimental approach, HDAC‐dependent *Kcnn1* regulation was studied by siRNA‐mediated inactivation of *Hdacs* in murine atrial HL‐1 cells. This approach revealed that *Hdacs* modulate *Kcnn1* expression in subtype‐specific manner, representing a major and novel finding of the present work. Among class I and class II HDACs studies, *Hdac9* inhibition specifically increased *Kcnn1* mRNA levels in HL‐1 cells. This is in contrast to redundant downregulation of *Kcnn1* transcripts that was similarly achieved by suppression of multiple *Hdacs* (i.e., *2*, *3*, *6*, *7*). Finally, the lack of *Kcnn1* regulation by *Hdacs* *1*, *4* and *5* indicates specificity of *HDAC*‐*KCNN1* interactions and argues against an unspecific class effect of *HDACs*. Differential regulation of *KCNN1* expression by *HDACs* significantly extends our current knowledge on cardiac electrophysiology and ionic remodeling in AF.

### 4.4 Physiological and clinical significance

Downregulation of transcripts encoding for repolarizing K_Ca_ potassium channels in RA may cause prolonged AERPs that are observed in this specific group of AF patients with HF (Lugenbiel et al., 2017; Schmidt et al., 2017). Furthermore, repolarization heterogeneity induced by *KCNN1* upregulation in LA in addition to its downregulation in RA tissue may generate a complex substrate for atrial arrhythmia that is refractory to antiarrhythmic therapy and poses a particularly severe therapeutic challenge. Furthermore, the novel link between K_Ca_2.1 channels and HDACs may serves as basis for mechanism‐based antiarrhythmic therapy. Antiarrhythmic concepts that specifically aim at correcting or preventing arrhythmia‐induced electrical remodeling may improve and personalize AF treatment. HF‐related atrial arrhythmogenesis is characterized by decreased atrial K^+^ currents, resulting in prolongation of atrial APD and AERP (Lugenbiel et al., 2017; Schmidt et al., 2017). Reduced K_Ca_ channel expression and function in AF may contribute to this electrophysiological hallmark of AF/HF patients (Diness et al., 2010; Li et al., 2009; Ozgen et al., 2007; Qi et al., 2014; Skibsbye et al., 2014; Tsai et al., 2016; Zhang, Timofeyev, et al., 2014). Reversal of K_Ca_2.1 channel remodeling could represent an individualized strategy for rhythm control in this specific AF patient entity. Specifically, modulation of HDAC expression and function could serve as upstream target for antiarrhythmic interventions. Translation of this epigenetics‐based paradigm requires future basic scientific assessments and proof‐of‐concept studies in large animal models that will be initiated based on the present findings.

### 4.5 Implications for ventricular arrhythmogenesis

In addition to its predominant expression in atrial tissue, K_Ca_2.1 has been detected in ventricular myocardium as well (Tuteja et al., 2005; Xu et al., 2003). Ventricular K_Ca_2.1 channels may contribute (together with K_Ca_2.2 and K_Ca_2.3) to apamin‐sensitive K^+^ currents that were described in rodents (Chua et al., 2011; Hsieh et al., 2013; Lee et al., 2013; Ni et al., 2013) and show increased levels in HF models (Hsieh et al., 2013; Lee et al., 2013). A role for K_Ca_2.1 in ventricular electrical remodeling is further supported by increased expression in a rat model of HF that was reversed by beta‐blockade (Ni et al., 2013). K_Ca_2.1 regulation by HDACs might impact ventricular electrophysiology in health and disease as well. Prolonged QTc intervals were observed among patients receiving HDAC inhibitors for anticancer treatment (Lkhagva et al., 2016; Rasheed et al., 2007). Prolongation of ventricular APD and QTc intervals by HDAC inhibitors could in part be due to reduction of K_Ca_2.1 expression mediated via HDACs *2*, *3*, *5*, *6*, and *7* that were here revealed to downregulate *KCNN1* transcript levels. In addition, it is important to note that effects of HDAC inhibitors on other cardiac ion channels (Kopljar et al., 2016; McKinsey, 2011; Xu et al., 2013, 2016) may contribute to APD and QTc changes.

### 4.6 Potential limitations and future directions

This study was designed to advance the current understanding of electrical K_Ca_2.1 (*KCNN1*) channel remodeling associated with epigenetic mechanisms underlying AF complicated by HF. We acknowledge that relatively small sample sizes due to the large animal model and limitations in patient tissue acquisition resulted in low statistical power. In addition to remodeling of *HDAC* expression and its effect on *KCNN1* mRNA levels, direct histone modification or other epigenetic mechanisms such as DNA hypermethylation lie beyond the scope of the present work and require investigation in future studies. Furthermore, protein expression could not be analyzed as selective, subtype‐specific anti‐HDAC antibodies are currently not commercially available. In addition, while relative expression changes provide information on regulatory mechanisms, total transcript abundances need to be considered as well to evaluate the physiological relevance of HDAC‐dependent K_Ca_2.1 (*KCNN1*) regulation. Finally, human and animal data were derived from a clinically relevant AF sub‐entity exhibiting HF. Differences in pathophysiology between the porcine model with a tachymyopathy phenotype and humans with severe ischemic or dilatative cardiomyopathy may account for differential *KCNN1* remodeling observed here. To establish whether these findings may be extended to AF patients with preserved cardiac function respective cohorts without HF need to be studied. Potential dose‐dependent cardiotoxic effects require careful assessment during the evaluation of HDAC inhibitors for clinical antiarrhythmic treatment.

## 5 CONCLUSION

Differential remodeling of atrial K_Ca_2.1 channel transcript levels in AF with concomitant HF and associated changes in HDAC expression represent a previously unrecognized mode of epigenetic regulation in cardiac electrophysiology. Enhancement of *Kcnn1* mRNA transcript levels by genetic inactivation of *Hdac9* or suppression of *Kcnn1* following knockdown of *Hdacs* *2*, *3*, *6*, and *7* provide the mechanistic basis for individualized management of distinct AF disease stages that are characterized by increased‐ or decreased K_Ca_2.1 channel abundance and inverse changes in atrial APD, respectively. The clinical efficacy of HDAC modulators for tailored antiarrhythmic interventions in AF/HF patients requires validation in translational and clinical approaches.

## CONFLICT OF INTEREST

A.K.R. reports educational support from Boston Scientific, Johnson & Johnson, Abbott, and Medtronic. D.T. reports receiving lecture fees/honoraria from Bayer Vital, Boehringer Ingelheim Pharma, Bristol‐Myers Squibb, Daiichi Sankyo, Medtronic, Pfizer Pharma, Sanofi‐Aventis, St. Jude Medical, and ZOLL CMS. P.L. reports receiving lecture fees from Bayer Vital and Pfizer Pharma and educational support from Boston Scientific and Johnson & Johnson. The remaining authors have reported that they have no relationships relevant to the content of this paper to disclose.

## AUTHOR CONTRIBUTION

A.K.R., D.T., and P.L. conceived the study and designed the experiments. A.K.R., T.W., D.G., M.E.M., M.W., F.E.T.A., T.H., S.S., T.W., P.M., and P.L. contributed to material preparation, experiments, and data collection. All authors contributed to data analysis and interpretation. D.T. wrote the first draft of the manuscript. All authors contributed to critical reviewing and editing of the manuscript. All authors read and approved the final manuscript.
